# Potassium channel-targeted therapy in reducing vascular complications in diabetes mellitus: novel insights

**DOI:** 10.3389/fphar.2026.1796678

**Published:** 2026-05-28

**Authors:** Jovana Rajkovic, Andjela Ribic, Luka Rekovic, Milos Gostimirovic, Ana Bukarica, Dusko Terzic, Ljiljana Gojkovic-Bukarica

**Affiliations:** 1 Institute for Pharmacology, Clinical Pharmacology and Toxicology, Faculty of Medicine, University of Belgrade, Belgrade, Serbia; 2 Clinic for Cardiology, University Clinical Centre of Serbia, Belgrade, Serbia; 3 Clinic for Gynecology and Obstetrics “Narodni Front”, University of Belgrade, Belgrade, Serbia; 4 Institute for Cardiovascular Diseases “Dedinje”, University of Belgrade, Belgrade, Serbia; 5 Clinic for Cardiovascular Surgery, University Clinical Centre of Serbia, Belgrade, Serbia

**Keywords:** blood vessels, diabetes mellitus, potassium channel openers, potassium channels, vascular smooth muscle

## Abstract

Potassium (K^+^) channels are the most diverse group of ion channels and are required for many physiological functions such as cell excitability and insulin secretion. They are widely distributed throughout the body, but the majority of their subclasses are found in blood vessels, more specifically in the vascular smooth muscle cells (VSMCs) and in the endothelial cells (ECs). In blood vessels, they participate in the regulation of proper vascular tone. Certain diseases may impair the structure and function of vascular K^+^ channels and decrease their expression in the blood vessels, causing improper blood flow through vital organs and vascular tone dysfunction. These changes lead to clinically significant diseases (hypotension, hypertension, atherosclerosis, cerebrovascular diseases, etc.), which increase total and cardiovascular mortality. Diabetes mellitus (DM) is a condition that significantly alters the structure and function of K^+^ channels in the blood vessels. These changes contribute to the development of common DM-associated chronic macrovascular and microvascular complications, which further increases morbidity and mortality. These complications are especially prominent in diabetes mellitus type 2 (T2DM). Moreover, other concomitant pathophysiological processes impair K^+^ channels, which accelerate the progression of DM. This profound bidirectional patophysiological connection between DM and vascular K^+^ channels may redirect future antidiabetic therapy to novel mechanisms that include modulation of these channels. This up-to-date review summarizes the contribution of DM-induced vascular K^+^ channel dysfunction to vascular complications of DM and future directions in the development of novel K^+^ channels-targeted therapy for vascular disorders.

## 1 Introduction

Potassium (K^+^) channels represent widely distributed types of ion channels in eukaryotes and are classified into several families and subtypes (Miller, 2000). Besides the cell membrane, K^+^ channels are located in the membrane of various cell organelles (like mitochondria, endoplasmic reticulum, nuclei, etc.), in the cells of different tissues and organs (Toller et al., 2000; Quesada et al., 2002). The archetype of the K^+^ channel consists of four transmembrane, pore-forming alpha subunits (Kuang et al., 2015).

Different diseases may change the expression or function of K^+^ channels (Shieh et al., 2000). At the same time, congenital mutations of genes encoding K^+^ channels may be the basis of some other diseases (some of them are commonly referred to as channelopathies) (Staruschenko et al., 2023; Welch and Chung, 2022). The diseases associated with primary dysfunction of K^+^ channels are systemic hypotension/hypertension, atherosclerosis, vascular dementia, diabetic cardiomyopathy, congenital hyperinsulinism, Beckwith-Wiedemann syndrome, Brugada syndrome, LQTc (long QT) syndrome, etc (Sanguinetti and Spector, 1997; Kalish et al., 2016). Based on that, structural or functional dysfunction of K^+^ channels may be both the cause and the consequence of different diseases.

K^+^ channels are integral membrane proteins that enable the selective passage of K^+^ ions through the cell membrane, maintaining the electrochemical balance and decreasing the membrane potential (Zhang et al., 2024). In blood vessels, K^+^ channels are located on both endothelial cells (ECs) and vascular smooth muscles cells (VSMCs) and participate in proper balance of vascular tone. Therefore, their dysfunction may progress to the development of spasms of blood vessels (Sobey, 2001). Besides vascular dуsfunction, the dysfunction of K^+^ channels on VSMC may stimulate cell proliferation and arterial hardening, which predispose to the development of other cardiovascular diseases (CVD) like hypertension, diabetes, and atherosclerosis (Dogan et al., 2019).

Diabetes mellitus (DM) is recognized as a widely spread non-communicable disease with increasing prevalence globally. Based on data provided by the World Health Organization (WHO), a number of people living with DM increased from 200 million in 1990 to 830 million in 2022 (https://www.who.int/news-room/fact-sheets/detail/diabetes), while the projections for 2050 claim up to 1.3 billion (Global, 2023). DM, especially type 2 (T2DM), has an impact on the quality of life and life expectancy as well as on the medical economy (Butt et al., 2024; Fuentes-Merlos et al., 2021). It represents an independent risk factor for the people with CVD, mostly due to DM-related vascular complications, which increases their overall mortality (Martín-Timón et al., 2014).

One-third of patients who require coronary artery bypass grafting (CABG) surgery have DM (González Santos and Castaño Ruiz, 2002). It is known that DM causes a sustained increase in the vascular tone, decreased vasodilatation, and endothelial dysfunction (e.g., the inability of ECs to produce physiologically sufficient amounts of endothelial hyperpolarizing factors like nitric oxide (NO) and endothelial-derived relaxing factors (EDRF)). These are the reasons why the patients with DM are at higher risk for intra- and postoperative vascular complications (such as spasms and stenosis of bypass grafts) and other surgical complications compared to the patients without DM (Liu et al., 2025; Bezdenezhnykh et al., 2020).

Over time chronic hyperglycemia accelerates vascular complications by different mechanisms: enhancing the production of reactive oxygen species (Caturano et al., 2025); damaging the endothelial layer; stimulating inflammation of the vascular wall; decreasing NO production (Giacco and Brownlee, 2010); and changing the expression or regulation of the vascular K^+^ channels (Yang and Yang, 2025). This review summarizes up-to-date information about the status and function of K^+^ channels in the diabetic blood vessels, their link with the vascular complications, and perspectives for the K^+^-channel-targeted therapy.

Identifying specific abnormalities in the structure or function of K^+^ channels in vascular diseases, such as hypertension, pulmonary hypertension, atherosclerosis, hypercholesterolemia, and DM, may serve as a novel direction for the development of targeted and more precise therapy (Sobey, 2001). During the decades, different modulators of K channels were tested *in vitro* on different blood vessels or adequate cell lines (VSMC and EC). There are numerous peptide toxins, synthetic molecules, or natural compounds (like resveratrol) that regulate the function of different K^+^ channels, and they are used as pharmacological tools for investigating pathophysiological conditions involving these channels (Dogan et al., 2019). However, the regulation of K^+^ channel activity is dynamic as it is influenced by different physiological stimuli in the organism (chemical, metabolic, genetic, or signaling/enzymatic). Also, the expression of K^+^ channels is highly tissue-specific, and certain clinical factors (sex, age, comorbidities) are very individual, which limits wider incorporation of K^+^ channels targeted therapy in clinical practice (Garrud et al., 2026).

K^+^ channels are potential targets for the development of cardioplegic solutions, more specifically for graft preservation in the CABG (Jahangir and Terzic, 2005). So far, the different PCOs (aprikalim, nicorandil, pinacidil, and diazoxide), as monotherapy or combined, have been tested as possible potent myoprotection and vasodilation agents for patients with heart or vascular diseases. However, an ideal solution for graft preservation has not been established yet, and there is only one clinically approved solution for vein preservation (Kiss et al., 2022). Specific solutions instead of common but less effective solutions (like physiological saline solution or heparinized autologous blood) may provide long-term success protecting the endothelial layer during the CABG (Bouhout et al., 2018). PCOs remain promising agents for future research in the development of these solutions.

The pharmacology of K^+^ channels has been extensively studied for the last 30 years in the Laboratory for Ion Channel Pharmacology, at the Institute of Pharmacology, Clinical Pharmacology, and Toxicology, Medical Faculty, University of Belgrade, Serbia. The main focus of this research group has been EC and VSMC K^+^ channels. From the first experiments performed on the isolated animal blood vessels (Gojkovic-Bukarica et al., 2008; Gojković and Kazić, 1994) to isolated human blood vessels of different origins (Stojnic et al., 2007; Jovanović et al., 1994), research has been expanded to different pathological conditions, such as T2DM, gestational DM, and pregnancy-induced hypertension. So far, different patterns of expression and function of the main classes of K^+^ channels have been demonstrated in different tissues (rat aorta, rat renal artery, rat portal vein, human umbilical vein, human internal mammary artery, human saphenous vein, human radial artery, etc.) as well as the influence of the disease on their expression (Djokic et al., 2020; Djokic et al., 2019; Rajkovic et al., 2024; Rajkovic et al., 2020; Djokic et al., 2023). Besides the effects of different potassium channel openers (PCOs), the experiments were further extended into researching the interaction between natural compounds such as polyphenols and the K^+^ channels in the VSMCs and ECs (Novakovic et al., 2006). After more than 30 years of experience in the field of vascular K^+^ channel pharmacology, our results confirm their physiological role in the healthy VSMC. At the same time, their dysfunctionality has been shown in diseases such as DM and hypertension.

Over the decades, K^+^ channels have been considered as a promising tool for the development of adequate intra-operative solutions for bypass graft storage, adjusting pharmacotherapy based on comorbidities (i.e., individualization of therapy), and targeting molecular spots for the development of new drugs. Differences in K^+^ channel expression and function between arteries, veins, and umbilical vessels allow precise local control of vascular tone and adaptation to specific physiological requirements. Understanding these mechanisms has significant clinical implications, especially for the development of selective pharmacological modulators that target K^+^ channels of specific vascular compartments without causing systemic effects (Batista et al., 2023).

## 2 Structure and function of K^+^ channels in blood vessels

K^+^ channels may be divided according to their structure (the number of transmembrane domains—TMDs), and activation mechanisms. According to the first classification, there are three subclasses of K^+^ channels: the ones with two, four, or six TMDs (Kuang et al., 2015). According to the second classification, K^+^ channels may be voltage-gated K^+^ (Kv) channels, calcium-activated K^+^ (K_Ca_) channels, inward rectifier K^+^ (Kir) channels, and two-pore domain K^+^ (K2P) channels (Li et al., 2022).

**FIGURE 1:**
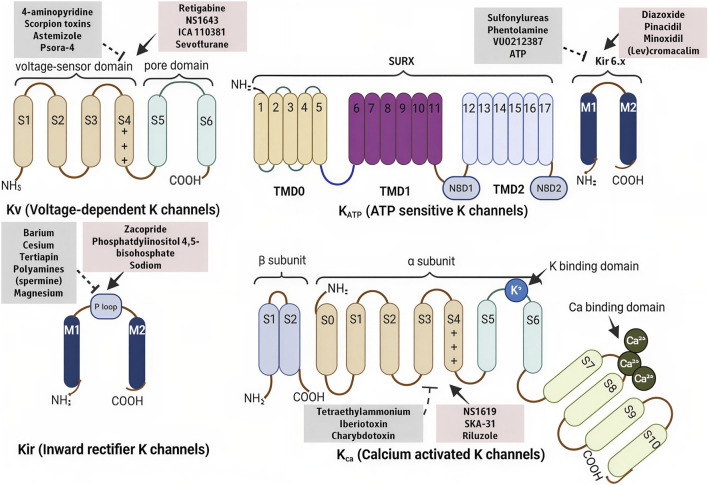
Basic structure of different types of K^+^ channels in VSMC and their pharmacological modulators. Note that the VSMC K_ATP_ channel consists of SUR2B and Kir6.1 subunits. The α subunit of K_Ca_ channels represents the voltage-sensitive domain, which is structurally Kv. NBD1 and NBD2 are nucleotide binding domains; TMD0, TMD1, and TMD2 are transmembrane domains. Specifically, N-terminal of BK_Ca_ channels (S0 domain) of α subunit is extracellular and modulates the activity of β subunit. Full arrows represent activators; dotted arrows represent inhibitors.

There are five primary classes of K^+^ channels in the blood vessels: K_Ca_, Kv, ATP-sensitive K^+^ (K_ATP_), Kir, and K2P channels (Jackson, 2017). However, since the real contribution of K2P channels in homeostasis of vascular tone is still under investigation, this review will cover the other four classes. The molecular structure of the reviewed K^+^ channels and their pharmacological modulators may be seen in Figure 1 and Table 1.

**TABLE 1:** Genes, cell expression and pharmacological modulators of vascular K^+^ channels.

Channel type	Channel subtype (vascular)	Gene (human)	Cell expression (VSMC and/or EC) in physiological conditions	Pharmacological activator	Pharmacological blocker
K_v_	Kv1.x (1.1–1.8)	KCNA1-7	Both (VSMC and EC)	**Retigabine**	4-AP, TEA, Dendrotoxin, Margatoxin
	Kv2.x (2.1–2.2)	KCNB1-2	VSMC	**Retigabine**	4-AP, TEA, Guangxitoxin
	Kv3.x (3.1–3.4)	KCNC1-4	VSMC	EX15	4-AP, TEA
	Kv4.x (4.1–4.3)	KCND1-3	VSMC	Neferine, allocryptonine	**Flecainide, Amiodarone**
	Kv5.x	KCNF1	VSMC (electrically ‘‘silent’‘)	No direct pharmacological activator	4-AP, TEA, Barium (Ba^2+^)
	Kv6.x	KCNG1-4	VSMC (electrically ‘‘silent’‘)	No direct pharmacological activator	Lorcainide, **Propafenone**
	Kv7.x (7.1–7.5)	KCNQ1-5	Both (VSMC and EC)	**Retigabine**, Flupirtine, Fasudil	XE991, Linopiridine
K_Ca_	BK_Ca_	KCNMA1	Both (VSMC and EC)	NS1619, Mallotoxin	Iberiotoxin, Charybdotoxin
	IK_Ca_	KCNN4	EC	SKA-31, DC-EBIO	TRAM-34, Senicapoc
	SK_Ca_	KCNN2-3	EC	SKA-31, NS309	Apamin, Dequalinium
K_ATP_	Kir6.1/SUR2B	KCNJ8/ABCC9	Both (VSMC and EC)	**Minoxidil**, **Diazoxide**, **Nicorandil**	Glibenclamide, Gliclazide
Kir	Kir2.x	KCNJ2	Both (VSMC and EC)	No direct pharmacological activator	Barium (Ba^2+^)
	Kir3.x (GIRK)	KCNJ3/6/9/5	EC (predominantly)	G-proteins, Ethanol	Terciapin
	Kir4.x	KCNJ1-16	EC	pH, Mg^2+^	**Verapamil**, Amiodarone
	Kir6.x	KCNJ8/KCNJ11	VSMC	**Minoxidil**, **Diazoxide**, **Nicorandil**	Glibenclamide, Gliclazide

TEA-Tetraethylammonium, 4-AP–4-aminopyridine. VSMC-vascular smooth muscle cells, EC-endothelial cells. Bold–agents with known clinical significance in cardiovascular diseases.

In the VSMC, opening of K^+^ channels leads to membrane hyperpolarization and vasodilatation, while closing of these channels leads to membrane depolarization and vasoconstriction (Jackson, 2000). Each subclass of K^+^ channels has specific molecular structure, regulation, and role in the vasoactivity mediated by VSMC or ECs. Individual subtypes are further divided into specific isoforms according to their different electrophysiological characteristics and tissue distribution (Werner and Ledoux, 2014).

**FIGURE 2:**
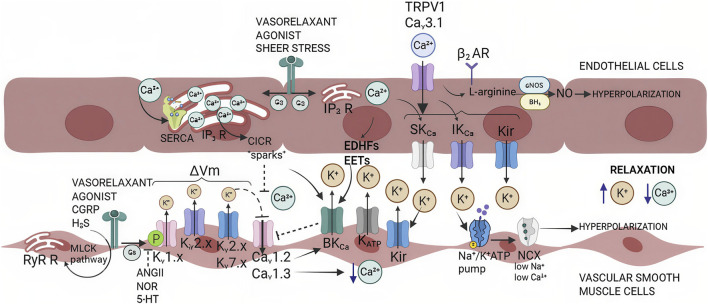
The function of K^+^ channels in blood vessels and their interaction in physiological conditions. Up: endothelial cells; down: VSMC. TRPV1 – transient receptor potential cation channels (vanilloid type), NCX–sodium-calcium exchanger, EDHFs–endothelium-derived hyperpolarizing factors, EETs–epoxyeicosatrienoic acid, MLCK–myosin light chain kinase, β_2_ AR - β_2_ adrenergic receptors, ANGII–Angiotensin II, 5-HT - serotonin, CGRP–calcitonin gene-related peptide, SERCA–sarcoplasmic/endoplasmic reticulum Ca^2+^-ATPase, CICR–calcium-induced calcium release, eNOS - endothelial NO synthetase, BH4 (tetrahydrobiopterin) – cofactor of eNOS, RyR R–ryanodine receptors, IP_3_ R–inositol 1,4,5 triphosphate receptors.

In vascular cells (ECs and VSMCs), different subclasses of K^+^ channels are expressed, which are activated by the different stimuli such as calcium (Ca^2+^) ions, NO, changes in voltage and pH, or changes in the cellular energy status (Figure 2) (Li and Yang, 2023). Healthy ECs have high expression of TRPV1 receptors that mediates Ca^2+^ influx. Increased concentration of intracellular Ca^2+^ in the ECs triggers Ca^2+^ release from the sarcoplasmic reticulum (Ca^2+^-induced Ca^2+^ release), stimulates synthesis of NO and other diffusible factors (EDHFs, EETs), and directly modulating the activity of endothelial SK_Ca_ and IK_Ca_. EDHFs, EETs and Ca^2+^ sparks directly stimulate BK_Ca_ channels in the VSMC. Ca^2+^ sparks, Kv (mostly Kv2.1) and BK_Ca_ channels inhibit VSMC Ca_v_1.2 channels, further limiting the Ca^2+^ influx into VSMC. The influx of K^+^ ions via SK_Ca_ and IK_Ca_ stimulate the activity of Na^+^-K^+^ pump which out more Na^+^ ions. Decreased intracellular Na^+^ concentration inhibits the activity of the NCX (Na^+^-Ca^2+^ exchanger) and decreases the concentration of intracellular Ca^2+^. All those processes promote hyperpolarization and relaxation of a blood vessel (see Figure 2).

Structurally, K^+^ channels contain a selective filter that precisely coordinates the passage of K^+^ ions, especially in relation to sodium (Na^+^) ions (Li J. et al., 2023). The selective filter consists of highly conserved sequence of amino acids known as TVGYG (Thr-Val-Gly-Tyr-Gly) that is responsible for the high selectivity of K^+^ ions compared to the Na^+^ ions (Doyle et al., 1998). Electrophysiologically, membrane depolarization activates the voltage-sensing domains of these channels, while a concurrent rise in intracellular Ca^2+^ concentration acts as an allosteric modulator. Ca^2+^ ions bind to high-affinity sites, such as RCK domains in BK_Ca_ channels or calmodulin-binding sites in SK_Ca_ channels, inducing conformational changes in the inner gate helices. These changes significantly increase the open probability of the channels, allowing a substantial K^+^ efflux even at sub-threshold membrane potentials (which typically ranges from −60 mV to −40 mV). The outward K^+^ current drives the membrane potential toward the K^+^ equilibrium potential, producing repolarization or hyperpolarization. This hyperpolarization, in turn, closes voltage-gated Ca^2+^ channels (VGCCs), terminating the initial Ca^2+^ influx and stabilizing the cellular resting potential. In VSMCs and ECs, such K^+^ channels generate so-called *spontaneous transient outward* currents, or stationary K^+^ currents, which directly affect the membrane potential and the contractile elements of the cell (Jackson, 2000).

The electrophysiological properties of K^+^ channels depend on their structure and genetic variability. Kv channels open at specific values of membrane potentials, with the S4 segment acting as a voltage-sensing element and the P-loop acting as a selective K^+^ filter (Barros et al., 2012). K_Ca_ channel activity depends on the intracellular Ca^2+^ concentrations and membrane potential (Orfali and Albanyan, 2023), while K_ATP_ channels link the current energy status of the cell with conductance, responding to the ATP/ADP ratio (Taskin et al., 2024). Kir channels help stabilization of the membrane potential at values below the K^+^ equilibrium, and some genetic mutations, for example, in the KCNJ2 gene, affect their activity (Hibino et al., 2010). Different alleles of the gene and beta-subunit modulate the kinetics of opening, inactivation, and voltage threshold of the K^+^ channels, which enables precise regulation of the membrane potential and vascular tone (Ko et al., 2008).

In humans, the genes encoding the different alpha-subunits of K^+^ channels are located on different chromosomes, reflecting the evolutionary diversification of the subclasses. For example, the gene KCNQ1 (Kv7.1) is located on chromosome 11p15.5-p15.4, the gene KCNH2 (Kv11.1) on chromosome 7q36.1, and the gene KCNMA1 (BK, K_Ca_1.1) on chromosome 10q22, while the subunits of K_ATP_ channels, KCNJ11 and ABCC8 are located on chromosome 11p15.1, and KCNJ8 and ABCC9, on chromosome 12p12.1 (more details may be seen in Table 1). Such chromosomal distribution enables different expression and regulation of K^+^ channels in vascular and other tissues, as well as modulation of their function (Pereira da Silva et al., 2022; Tykocki et al., 2017).

### 2.1 Voltage-gated K^+^ (Kv) channels

Voltage-gated K^+^ (Kv) channels respond to the changes in the voltage of the cell membrane. They are activated upon depolarization, enable the efflux of K^+^ ions from the cell, and cause hyperpolarization (Grizel et al., 2014). This reduces the activation of voltage-dependent Ca^2+^ channels, and this all together leads to vasorelaxation (Ko et al., 2008). Structurally, each α-subunit of a typical Kv channel has 6 transmembrane helices (S1-S6), of which the S4 segment carries amino acids with positive residues (arginine) and acts as a sensing segment for voltage change and eventually triggers conformational changes that open or close the channel. Its structure also includes an internal C-terminus and N-terminus, which allow interaction with β-subunits and cytosolic signal molecules, modulating channel conductance and its kinetics (Huang et al., 2023). Constituents of a cell membrane and internal mediators participate in the creation of macromolecular complexes between Kv channels in VSMC and transient receptor potential (TRP) channels, L-type Ca channels, regulatory proteins such as kinases and phosphatases, and scaffold proteins (Pereira da Silva et al., 2022). A change in the lipid composition of the membrane (distribution of phospholipids, phosphatidylinositol 4,5-bisphosphate (PIP_2_), or cholesterol) can shift membrane voltage thresholds at which the Kv channel opens, thereby modulating its activation (Kasimova et al., 2014). For example, increased PIP_2_ levels typically facilitate opening of the Kv channel, whereas elevated cholesterol concentration potentiates its closed state and decreases the sensitivity of these channels to depolarization (Zheng et al., 2011). Interestingly, it has been demonstrated that only margatoxin-sensitive VSMC K^+^ channels (Kv1.1 and Kv1.2 in the VSMC and Kv1.3 in the ECs) are involved in the resveratrol-induced vasorelaxation of the diabetic rat renal artery, compared with a non-diabetic renal artery, where other subtypes of K^+^ channels were registered (Gojkovic-Bukarica et al., 2019). Increased expression of Kv1.3 in diabetic blood vessels is confirmed by molecular techniques, and those channels represent a potential pharmacological target for treating DM (Choi and Hahn, 2010).

Among the subtypes of Kv channels, Kv7 channels are most abundant in the vascular cells, especially in the VSMCs of arterioles, where they maintain membrane resting potential and restrict excessive depolarization (Tsai et al., 2020). Recent studies have identified a specific subtype, Kv7.5, as a key channel that enables the connection of signals, lipids, and metabolites from perivascular adipose tissue with the tone of VSMC and regulation of blood pressure and vascular resistance (Gollasch et al., 2025). So far, several specific molecules have been identified that act as the activators of Kv7 channels, for example, flupirtine and retigabine (ezogabine), originally developed as analgesics and antiepileptics but later recognized also for their potential to promote vasodilatation through the opening of vascular Kv7 channels (Joshi et al., 2009). However, their wider clinical application in cardiovascular pathology is limited due to serious hepatotoxicity, which is why they have been withdrawn or strictly restricted in many countries (Wurm et al., 2022; Stott et al., 2014). At the same time, new, more selective Kv7 activators such as the experimental compounds URO-K10, ML213, and ICA-069673 are being developed, which show encouraging results in preclinical phases but still without a confirmation in clinical trials (Villegas-Esguevillas et al., 2023; Mondéjar-Parreño et al., 2020; Lee et al., 2020).

Investigating other specific Kv channel subtypes in different vascular regions, such as resistance arterioles, coronary arteries, or cerebral arteries, and their regulation by lipoproteins, adipokines, oxidative stress, and signaling kinases still represents an active area of research, tending to develop new drugs to treat vascular dysfunction with a better safety profile.

### 2.2 ATP-sensitive K^+^ (K_ATP_) channels

ATP-sensitive K^+^ (K_ATP_) channels were first described in cardiac cells by Noma in 1983 (Noma, 1983). K_ATP_ channels are essential regulators that connect the energy status of the cell with the electrical membrane potential and vascular tone (Foster and Coetzee, 2016). They close when the concentration of intracellular adenosine triphosphate (ATP) is high and open when the ATP/ADP (adenosine diphosphate) ratio is reduced, thus acting as metabolic sensors of the cells. Structurally, K_ATP_ channels are octameric complexes composed of four Kir6.x subunits, which form the ion pore, and four regulatory SUR (sulfonylurea receptor) subunits that regulate channel opening (Daoud et al., 2024). In VSMC, the most prevalent subtype of K_ATP_ channels is the Kir6.1/SUR2B isoform, while in the EC it is the Kir6.2/SUR2B isoform (Aziz et al., 2017). The SUR subunits of the K_ATP_ channel function as a central modulatory junction that integrates signaling from various pharmacological agents and endogenous ligands (Patton et al., 2024). Pharmacological agents targeting the SUR subunit include blockers like gliclazide, glimepiride, repaglinide, and mitiglinide, which promote channel closing, and activators like cromacalim and nicorandil, which promote channel opening (Li et al., 2024; Sung et al., 2021). In addition to these external modulators, defects in SUR subunits disrupt their interactions with membrane lipids, such as PIP_2_ and cholesterol, leading to local lipid redistribution that destabilizes the open state of the channel (Patton et al., 2024). Furthermore, altered SUR function modifies the channel’s responsiveness to oxidative metabolites and signaling proteins, including kinases and phosphatases, thereby directly affecting channel conformation and shifting the activation threshold in the VSMCs (Sung et al., 2021). Endogenous liposoluble molecules from the perivascular tissue and second messengers, such as cyclic adenosine-monophosphate (cAMP) and cyclic guanosine-monophosphate (cGMP), can also modulate SUR subunits, thereby adjusting channel opening in accordance with metabolic and chemical needs (Li et al., 2024).

Recent cryogenic electron microscopy studies have elucidated that SUR2B contains specific domains that mediate interactions between ATP-binding sites and Kir6.1 subunits, allowing fine-tuning of the channel sensitivity to magnesium-ADP and ATP. Specifically, conformational changes in SUR2B upon nucleotide binding are transmitted to Kir6.1 via inter-subunit interfaces, stabilizing either the open or closed state of the channel depending on the ATP/ADP ratio (Driggers and Shyng, 2023). This mechanism allows K_ATP_ channels to respond quickly to changes in cellular metabolism and open under stressful conditions such as hypoxia, ischemia, or metabolic acidosis (Sung et al., 2021).

The Kir6.x subunits are encoded by 2 different genes that share 71% of identity in the amino acid composition, while the SUR subunits (SUR1, SUR2A, and SUR2B) are encoded by two different genes, considering that SUR2A and SUR2B are the splice variants of the same gene. It is important to emphasize two important properties of the SUR subunit: 1) it determines the specificity and selectivity of the K_ATP_ channel agonists and antagonists, and 2) it shows selective, tissue-dependent distribution (Liu et al., 2001; Matsuo et al., 2000; Ammälä et al., 1996).

SUR1 is expressed at higher levels in pancreatic islets and in the brain. SUR2A shares 68% amino acid identity with SUR1 and has a low affinity for glibenclamide, while SUR2B differs from SUR2A by 42 amino acids in the C-terminus (where it is, instead, similar to SUR1). The SUR2A is expressed predominantly in heart and skeletal muscle, while SUR2B is expressed in VSMC. Today, there are several classes of antidiabetic drugs in clinical use, but sulfonylureas remain the most widely used in the treatment of T2DM (Sola et al., 2015). There are two proposed sites (site A and site B) modeled for the interaction between sulfonylureas, glinides, and SUR (Seino et al., 2012). Based on these different sites of interaction, sulfonylureas and glinides are divided into three groups. The first includes nateglinide, tolbutamide, and gliclazide, which bind specifically to the A site of SUR1, while the second group, which includes glimepiride and glibenclamide, binds non-specifically to the B sites of both SUR1 and SUR2A, as well as the A site of SUR1. The third group, which includes meglitinide and repaglinide, binds to the B site of SUR1 and SUR2A (Sola et al., 2015). Based on this model of interaction between the SUR subunit and sulfonylurea drugs, it has been proposed that this class of drugs has no impact on VSMC, which contain K_ATP_ channels with the SUR2B subunit (like K_ATP_ channels in VSMC). However, over the years, some extra-pancreatic actions of sulfonylurea drugs have been reported, including increased cardiac contractility, effects on water balance (either diuretic or anti-diuretic), and inhibition of platelet aggregation (Lebovitz and Feinglos, 1978). Despite the conflicting results of clinical trials on the effects of sulfonylureas on cardiovascular safety, the development of other drug classes began, for example, more selective PCOs, like iptakalim, for the treatment of hypertension. It has been shown that some of the antidiabetic agents (e.g., rosiglitazone and phenformin) also act as VSMC K_ATP_ channel inhibitors as well (Shi et al., 2012). These antidiabetic agents are not categorized in the sulfonylurea family.

Under physiological conditions, the opening of K_ATP_ channels in VSMC causes the efflux of K^+^ ions from the cell, membrane hyperpolarization, and inhibition of voltage-dependent Ca^2+^ channels, which decrease intracellular Ca^2+^ ions and cause vasodilatation (Aziz et al., 2014). In this way, K_ATP_ channels participate in local autoregulation of blood flow in metabolically high-active tissues. In cerebral arteries, they increase cerebral blood flow in hypoxia or other states with depleted ATP production (Daoud et al., 2024).

Diseases significantly alter the expression and function of K_ATP_ channels (Djokic et al., 2019; Liu et al., 2016). A recent study on human umbilical arteries from women with preeclampsia reported a reduction in K_ATP_ channel expression and reduced vasodilatation, suggesting their role in hypertensive disorders in pregnancy (Zampieri et al., 2024). In the human internal mammary artery and the human saphenous vein, commonly used bypass grafts, the presence of T2DM decreased the levels of Kir6.1 (in the artery) and SUR2B (in the vein) in VSMC (Rajkovic et al., 2024; Rajkovic et al., 2020). In contrast, gain-of-function mutations of genes encoding K_ATP_ channels, as seen, for example, in Cantu syndrome, result in permanent vasodilatation, hypotension, and vascular anomalies (Huang et al., 2018). These conditions confirm that K_ATP_ channels have bidirectional physiological and pathophysiological roles and that both excessive and insufficient channel activity disturb the finely balanced vascular tone.

The K_ATP_ channels represent potential therapeutic targets. Pharmacological activators, like diazoxide, pinacidil, or levcromakalim, have shown anti-vasoconstrictive properties in experimental models of hypertension (Adebiyi et al., 2008). However, due to systemic effects, especially in the myocardium and pancreas, the development of selective modulators of VSMC K_ATP_ channels is becoming a priority (Li et al., 2024). Moreover, there are an entire group of pharmacological agents collectively referred to as PCOs, which primarily act as activators of K_ATP_ channels. These compounds represent chemically diverse classes that share a common mechanism—the facilitation of K^+^ efflux through K_ATP_ channels, membrane hyperpolarization, and relaxation of VSMC (Edwards and Weston, 1990; Brayden, 2002). Beyond their vascular effects, PCOs also show cardioprotective (diazoxide and cromakalim) (Wang et al., 2026), neuroprotective (cromakalim and nicorandil, both tested on T1DM rats) (Pithadia et al., 2017), and beneficial metabolic effects (diazoxide, the only Food and Drug Administration (FDA)-approved drug for congenital hyperinsulinism) (Brar et al., 2020). Due to the above, PCOs remain objects of researchers’ interest, particularly those who tend to design vascular tissue-selective modulators (Nichols, 2023; Lv et al., 2022).

There are several PCOs recently investigated in different clinical trials. A novel small-molecule selective PCO of Kv7.2/Kv7.3 channels, XEN1101, was investigated in the treatment of focal-onset seizures (FOSs) in phase IIb randomized, double-blind, placebo-controlled clinical trials (French et al., 2023). The results are promising, supporting further clinical development of XEN1101. Later it was labeled as “azetukalner,” a novel and potent opener of Kv7 channels. It was investigated for the treatment of adults with major depressive disorders (MDD), epilepsy, and other neurological disorders. In a multicenter, proof-of-concept, phase 2, randomized, double-blind, parallel-group, placebo-controlled clinical trial, preliminary findings supported its further clinical development for the treatment of MDD (Butterfield et al., 2025).

Also, some of the old PCO drugs were investigated for the new indications. The combination of coronary reperfusion therapy and infusion of nicorandil, which is marked as a hybrid of PCO (mitoK_ATP_-specific opener) and nitrate, improves the left ventricular function in patients with acute myocardial infarction (Miura and Miki, 2003). Additionally, chronic treatment with nicorandil significantly improved the prognosis of patients with high-risk stable angina pectoris. However, based on basic research, the protective function of PCOs (K_ATP_ channel openers) is compromised by concurrent hypercholesterolemia and administration of sulfonylureas for DM. In the recent clinical trial, nicorandil showed improvement on one-lung ventilation (OLV)-induced pulmonary injury in 60 patients. The results from these trials suggested that nicorandil acted on mitoK_ATP_ via PI3K/Akt to reduce apoptosis in the operated lung.

In the interventional single-center clinical trial, the role of NN414, a selective K_ATP_ channel opener for the Kir6.2/SUR1 channel subtype in neurons and β-pancreatic cells, was investigated in adult patients (both males and females) with migraine. The data from the trial support the notion that the Kir6.1/SUR2B subtype, rather than the Kir6.2/SUR1, is a key channel in the signaling pathways that trigger migraine attacks (Kokoti et al., 2024). These findings highlighted the development of selective Kir6.1/SUR2B antagonists as a strategy for treating migraines.

However, some of the PCOs failed to exhibit superior efficacy during the later phase of clinical research. That was the case with BMS-204352, the effective opener of two important subtypes of neuronal K^+^ channels, BK_Ca_ channels and Kv7, initially considered for the treatment of stroke (Jensen, 2002).

### 2.3 Inward rectifier K^+^ (Kir) channels

Inward rectifier K^+^ (Kir) channels are a special group of K^+^ channels that enable the influx of K^+^ ions into the cell (Li and Yang, 2023). In the vascular tissue, these channels are in both ECs and VSMCs, where they maintain electrophysiological stability and respond to changes in extracellular K^+^ concentration (Sancho et al., 2022).

Structurally, Kir channels are tetramers, composed of four subunits each containing two transmembrane alpha-helices (M1 and M2) and an intersegment P-loop that forms the ion pore (Jogini et al., 2023). The large intracellular N- and C-terminal domains enable regulation by membrane lipids and regulatory proteins (Jogini et al., 2023). It is known that PIP_2_ stabilizes the open state of the channel, while cholesterol and intracellular polyamines, for example, spermine, block the efflux of K^+^ ions during depolarization, resulting in a characteristic inward-rectifier profile (Sancho et al., 2022; Jogini et al., 2023). Subtypes Kir2.1 and Kir2.2 are dominant in VSMCs, while Kir2.x channels predominate in the ECs (Li and Yang, 2023). Their activation maintains the negative membrane potential and mediates vasodilatation caused by an increase in extracellular K^+^ ions, prostaglandins, and increased blood flow (Fancher et al., 2020). In cerebral arteries, Kir channels function as flow sensors that transform mechanical shear stress into electrical signs of endothelial hyperpolarization and VSMC relaxation (Do Couto et al., 2024). The extent of hyperpolarization is influenced by the mechano-sensitivity of the Kir2.x channels (Sancho et al., 2022).

Interactions of Kir channels with protein complexes, including caveolins, syntrophins, and cytoskeletal elements, are important for their proper localization and functional association with the other ion channels (Hager et al., 2021). Therefore, Kir channels act as amplifiers of hyperpolarizing signals. Even small changes in potential caused by the activation of other K^+^ channels can be amplified by Kir-mediated influx of K^+^ ions, which hyperpolarizes VSMCs, reduces voltage-gated Ca^2+^channel activity, and ultimately lowers vascular tone (Do Couto et al., 2024).

Dysregulation of Kir channels is associated with numerous diseases. For example, a decrease in the functional expression of Kir2.1/Kir2.2 channels in ECs and VSMCs (commonly seen in hypercholesterolemia and hypertension) reduces vasodilatation and increases vascular reactivity in different blood vessels, like mesenteric resistance arteries, cerebral arterioles, pulmonary arteries, and subcutaneous adipose microvessels, leading to local or systemic hypertension (Ahn et al., 2022). In the cerebrovascular system, vasoconstriction may increase the risk for stroke, post-stroke cognitive impairment, CADASIL (Cerebral Autosomal Dominant Arteriopathy with Subcortical Infarcts and Leukoencephalopathy), Alzheimer’s disease, and other types of vascular dementia (Ahn et al., 2022). The major cause of the Kir2.1 channel dysfunction is PIP_2_ depletion, whose profound hydrolysis by the phospholipase C is especially prominent in people with diabetes. Similarly, high cholesterol concentrations lead to Kv2.1 channel suppression by enhancing plasma membrane rigidity and lowering the functional availability of PIP_2_ (Earley and Kleinfeld, 2021; Heydari et al., 2025). This change is one of the reasons why people with DM are at higher risk for cerebrovascular diseases, especially if they have associated hypercholesterolemia. On the contrary, in certain cerebrovascular diseases linked with chronic ischemia and increased metabolic demands (e.g., ischemic stroke, perinatal/neonatal injuries), upregulation of Kv2.1 channels is found (Yamamura et al., 2018), which may be one of the compensatory mechanisms for achieving proper blood flow and limiting further neuronal damage.

Based on all of the above, changes in the activity of Kir channels in VSMC and EC in different vascular beds may play both restrictive and protective roles, depending on the current metabolic state in the cells.

### 2.4 Calcium-activated K^+^ (K_Ca_) channels

Calcium-activated K^+^ (K_Ca_) channels determine vascular electrophysiology and vascular tone, as they link changes in the intracellular concentration of Ca^2+^ ions with the cell membrane potential. As previously stated, an increase in cytosolic Ca^2+^ activates these channels and allows efflux of K^+^ ions, which reduces membrane potential of the cell, closes voltage-dependent Ca channels, and relaxes blood vessels (Pereira da Silva et al., 2022).

Based on conductance and molecular structure, K_Ca_ channels are divided into three main groups: channels of high (Sola et al., 2015) conductance (BK, KCa1.1), medium (intermediate) conductance (IK_Ca_, K_Ca_3.1), and small conductance (SK, K_Ca_2.x) (Dong et al., 2016). Structurally, they are all tetrameric proteins with transmembrane domains and cytoplasmic regions with Ca^2+^-binding sites. BK_Ca_ channels have intrinsic sensitivity to Ca^2+^ and voltage changes, while SK_Ca_ and IK_Ca_ channels are activated upon binding of calmodulin, which acts as a constitutive cofactor (Nam et al., 2023).

In VSMCs, BK_Ca_ channels are dominant and function as a negative feedback loop in the control of vascular tone and are activated by the local releases of Ca^2+^ from the sarcoplasmic reticulum (Ca^2+^ sparks). This fine-balancing mechanism between an increase in cytosolic Ca^2+^ concentrations and a decrease in cytosolic K^+^ concentrations enables the stability of vascular contraction and prevents excessive depolarization (see Figure 1) (Taylor et al., 2023).

In ECs, K_Ca_3.1 and K_Ca_2.3 channels participate in the generation of EDH and NO-independent vasodilatation (Vera et al., 2023). Activation of these channels increases K^+^ efflux and causes propagation of a hyperpolarizing wave to VSMC via gap junctions (Li J. J. et al., 2023). In addition, EDH-dependent activity of K_Ca_ channels promotes efflux of Ca^2+^ into ECs and activation of endothelial nitric oxide synthase (NOS) and NO production (Vera et al., 2023).

The regulation of K_Ca_ channels is complex and includes phosphorylation (protein kinase A, G, and C), oxidative modifications, and changes in the composition of membrane lipids, as well as interactions with beta-subunits and protein scaffold structures (Van et al., 2024). Besides, these channels may be modulated by many hormones, mediators, and metabolic signals, such as hydrogen peroxide (H_2_O_2_) and cGMP, as well as mechanical stress, all of which adjust vascular tone to current physiological needs (Taylor et al., 2024).

K_Ca_ channels represent fine-regulatory points of vascular homeostasis. Disturbances in K_Ca_ channel function are associated with endothelial dysfunction, hypertension, DM, and atherosclerosis (Vera et al., 2023). In diabetic models, a decrease in the expression of K_Ca_3.1 and K_Ca_2.3 was recorded, resulting in impaired EDH-dependent dilatation (Mishra et al., 2021). Conversely, pharmacological activators, such as SKA-31 and NS309, have shown the ability to restore endothelial function and improve microvascular flow (Mishra et al., 2026).

In the last 2 years, SKA-31 has been recognized as a leading selective activator of SK_Ca_ and IK_Ca_ channels. Acting primarily in ECs, SKA-31 enhances Ca^2+^-induced opening of K_Ca_ channels and hyperpolarization (John et al., 2020). Preclinical research shows that long-term administration of SKA-31 improves endothelial function in the aorta of mice prone to atherosclerosis, as well as endothelium-dependent vasodilatation of rat mesenteric arteries in the diabetic models (Mishra et al., 2026). However, long-term activation of SK_Ca_ and IK_Ca_ channels by these agents may carry potential risks, for example, excessive vasodilatation, hypotension, or hypovolemic shock (Damkjaer et al., 2012). Moreover, unwanted effects are possible in other tissues where K_Ca_ channels are expressed, such as the heart, brain, and urinary tract (reduced responsiveness of coronary arteries to vasopressors, increased vascular permeability of the blood-brain barrier, and bladder ischemia) (Van et al., 2024). To combat and to prevent this issue, compensatory changes in other ion channels may happen (e.g., downregulation of Kir2.1 channels and upregulation of Na/TRP channels), which return the cell to its baseline membrane potential (Zuo et al., 2017). Finally, the safety, optimal dosing, and long-term efficacy of vascular K_Ca_ activators in humans remain to be thoroughly investigated, highlighting the need for careful translational studies before clinical application.

## 3 Type of K^+^ channels in the arteries, veins, and umbilical blood vessels

The distribution and functional roles of different subtypes of K^+^ channels in the vascular system show marked differences between arteries, veins, and umbilical vessels. These differences reflect the specific hemodynamic requirements and mechanisms of wall tone regulation in each of the blood vessel types.

In arteries, especially resistive and muscular ones, all four subtypes of reviewed K^+^ channels (Kv, K_Ca_, Kir, and K_ATP_) are present (Nelson and Quayle, 1995). Kv channels, mainly Kv1.x and Kv7.x, enable membrane potential and prevent excessive depolarization and contraction of VSMCs (Jackson, 2018). K_Ca_ channels, mainly BK_Ca_ (K_Ca_1.1), act as the negative feedback between intracellular Ca^2+^ sparks and the membrane, enabling fine control of vascular tone (Pereira da Silva et al., 2022). In arterial ECs, K_Ca_3.1 (IK_Ca_) and K_Ca_2.3 (SK_Ca_3) channels contribute to EDH and signal transmission to smooth muscle (Li J. J. et al., 2023). K_ATP_ channels link the metabolic status of the cell with vasodilatation, especially during hypoxia or reduced ATP levels, while Kir channels (Kir2.x) serve as amplifiers of hyperpolarization signals caused by a local increase in the extracellular concentrations of K^+^ ions (Pereira da Silva et al., 2022).

Dominant subtypes of K^+^ channels in veins are K_Ca_ and K_ATP_ channels, which allow venodilatation and adequate venous return (Rajkovic et al., 2020). Although studies on K^+^ channels in the veins are less common than those on the arterial K^+^ channels, it is known that reduced function of K_Ca_ channels in the venous endothelium increases vascular tone and leads to chronic venous insufficiency (Raffetto and Khalil, 2021). K_ATP_ channels in venous smooth muscles mediate metabolic relaxation during hypoxic and stressful conditions, maintaining flow and preventing spasms (Rajkovic et al., 2020).

In the umbilical blood vessels, tone regulation relies almost exclusively on local biochemical signals, because these vessels are not innervated by the sympathetic nerves. In the human umbilical artery, BK_Ca_ (K_Ca_1.1), Kv, and Kir channels are dominant (Zampieri et al., 2024; Lorigo et al., 2020). Also, the presence of K_Ca_3.1 and K_Ca_2.3 channels is noted, although their functional role is still under research (Zampieri et al., 2024). K_ATP_ channels are less expressed but may contribute to vasodilatation in hypoxia or acidosis (Zampieri et al., 2024). In the human umbilical vein, Kv and BK_Ca_ channels regulate passive relaxation and adjust fetal venous outflow to changes in fetal-placental flow (Rojas et al., 2020). In certain conditions like pregnancy-induced hypertension, a decreased expression of Kv1.3 channels was observed (Djokic et al., 2020). Lipophilic modulators such as farnesol have recently been shown to induce relaxation of umbilical arteries by activating multiple types of K^+^ channels, further confirming their integrative role in fetal circulation (Batista et al., 2023). Additionally, it has been shown that the red wine polyphenol resveratrol induces vasodilatation of the human umbilical vein by modulating different K^+^ channels (Rojas et al., 2020; Protić et al., 2015; Protić et al., 2014).

## 4 Impact of DM on the vascular K^+^ channels

DM, especially T2DM, predisposes to both micro- and macrovascular complications, including retinopathy, nephropathy, neuropathy, atherosclerosis, and coronary and peripheral vascular disease (Lu et al., 2023). The majority of them are consequences of K^+^ channel dysfunction (Yang and Yang, 2025).

Diabetic retinopathy involves retinal vascular dysfunction, edema, and neovascularization. K^+^ channels, especially Kir4.1 in Müller cells, maintain K^+^ balance and proper membrane potential (Kofuji et al., 2000). In hyperglycemia, reduced expression of Müllerian Kir4.1 channels leads to intracellular accumulation of K^+^ and water (Li et al., 2021). The intracellular edema of Müller cells exerts mechanical stress on surrounding structures, including the basal membranes of retinal capillaries, impairing the integrity of the blood–retinal barrier (BRB). This disruption increases vascular permeability, allowing fluid and plasma components to leak into the retinal extracellular space, which further worsens the diabetic retinopathy (Yang et al., 2022). Also, variants of KCNJ11 (K_ATP_ channel) are associated with an increased risk of diabetic retinopathy (Morgante et al., 2025). Reduced function of Kir and other K^+^ channels disrupts retinal perfusion and increases oxidative stress and inflammation, which accelerate retinal vascular damage (Li et al., 2021). Although data on retinal Kv and K_Ca_ channels are limited, disturbances in these channels in DM may indirectly worsen vascular tone and perfusion (Yang et al., 2022). Targeted modulation of K^+^ channels represents a potential therapeutic direction to preserve retinal function and prevent the progression of diabetic retinopathy.

In both diabetic animals and humans, BK_Ca_ channels in peripheral resistance arteries show reduced sensitivity to Ca^2+^ ions, decreased opening probability, and a connection between alpha and beta subunits, which increases vascular tone (Nieves-Cintrón et al., 2017). Specifically, arteries of patients with DM show a decrease in the amplitude of spontaneous BK_Ca_ currents and reduced opening frequency, without significant changes in the total amount of subunits, which implies that the functional modifications are more prominent than a simple change in the expression. Also, the reduced response to BK_Ca_ activators in resistance arteries in DM models indicates that the channel, although present, is incapable of achieving vasodilatation during increased metabolic stress.

Reduced functionality of K_ATP_ channels in VSMC in the DM model decreases vasodilatation in hypoglycemic or hypoxic conditions, making preservation of blood perfusion in stressed tissues more difficult (Li et al., 2015a). In arteries from DM models, administration of K_ATP_ agonists decreased relaxation more than in the control groups, suggesting that K_ATP_ channels are not adequately responsive. Dysregulation of these channels enhances vascular stress during phases when an adaptive response is required.

Kv channels are also altered in DM. A recent review highlights that remodeling of Kv and Kir channels in ECs and VSMCs in diabetic patients enhances vascular reactivity and susceptibility to oxidative stress and inflammation, which accelerates atherogenesis and vascular rigidity (Yang and Yang, 2025).

In microangiopathies, especially in the kidneys, altered K^+^ channels and consequent imbalance in medial perfusion damage the filtration barrier (Guo et al., 2024). Generation of free oxygen radicals and proinflammatory mediators follows this damage, which further disrupts K^+^ channel homeostasis. In a recent article, blockers of certain K^+^ channels have shown potential to ameliorate diabetic renal injury (Guo et al., 2024).

Finally, remodeling of VSMC K^+^ channels in the DM affects the fine adjustment of the vascular tone to the current tissue metabolic demands (Yang and Yang, 2025).

**FIGURE 3:**
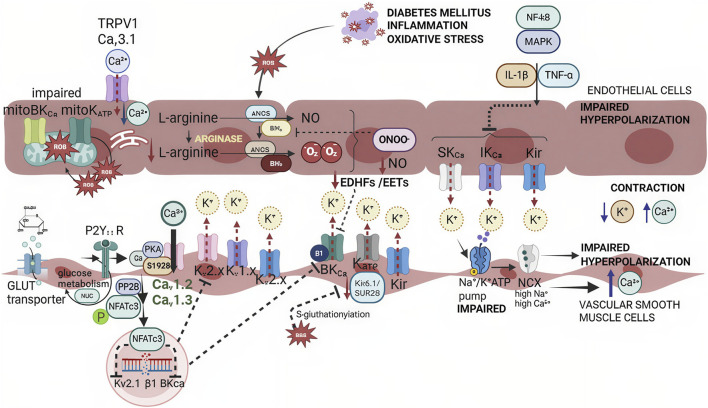
The function of potassium channels in blood vessels and their interaction in pathological conditions (T2DM, oxidative stress, inflammation). Up: endothelial cells, down: VSMC. TRPV1 – transient receptor potential cation channels (vanilloid type), NCX–sodium-calcium exchanger, EDHFs–endothelium-derived hyperpolarizing factors, EETs–epoxyeicosatrienoic acid, MLCK–myosin light chain kinase, β_2_ AR - β_2_ adrenergic receptors, ANGII–Angiotensin II, 5-HT - serotonin, CGRP–calcitonin gene-related peptide, SERCA–sarcoplasmic/endoplasmic reticulum Ca^2+^-ATPase, CICR–calcium-induced calcium release, eNOS - endothelial NO synthetase, BH4 (tetrahydrobiopterin) – cofactor of eNOS, RyR R–ryanodine receptors, IP_3_ R–inositol 1,4,5 triphosphate receptors, NFaTc3 – nuclear factor of activated T lymphocytes.

Hyperglycemia, oxidative stress, and chronic inflammation affect indirectly the function of different types of K^+^ channel in VSMC (Nieves-Cintrón et al., 2021) (Figure 3).

Figure 3 depicts the following changes. In ECs, L-arginine is converted by eNOS into nitric oxide (NO), which promotes vasodilation. Damaged ECs show decreased expression of TRPV, which limits the Ca^2+^ influx. As a consequence of reduced concentrations of BH_4_, L-arginine, and diffusible factors, the NO synthesis is impaired, while the byproduct of this process, peroxynitrite (ONOO-), further worsens oxidative stress (Pacher et al., 2007). Mitochondrial channels (mitoBK_Ca_, mitoK_ATP_) are also dysfunctional, increasing ROS production. Inflammatory mediators, TNF-α, and IL-1β inhibit the activity of SK_Ca_ and IK_Ca_ on the vascular endothelium (Félétou, 2009). In VSMC, increased concentrations of nucleotides made in enhanced glucose metabolism bind to P2Y_11_ receptors, which are coupled with the cAMP-PKA pathway. After activation, the Cav1.2 channel is phosphorylated at the serine in place of 1928. This increases its function and the Ca^2+^ ion influx. On the other hand, the expression of the Kv2.1 β1 subunits of BK_Ca,_ K_ATP_ (Kir6.1/SUR2B), and Kir channels is decreased. The activity of the Na^+^-K^+^ pump is impaired, pumping out less Na^+^, which increases the concentration of intracellular Ca^2+^. Other regulators shown include NFATc3, SERCA, RyR, IP_3_ receptors, and GLUT-mediated glucose metabolism. Overall, oxidative stress (via NF-κB, MAPK, TNF-α, IL-1β) disrupts NO/ROS balance, impairs functionality of K^+^ channels, increases intracellular Ca^2+^, and promotes vasoconstriction (Harvey and Hell, 2013; Shattock et al., 2015).

DM amplifies the imbalance between the NO/ROS ratio and stimulates oxidative stress production. High generation of ROS intermediates modifies protein domains of K^+^ channels or their cofactors and reduces their sensitivity to Ca^2+^, ATP, or voltage (Yang and Yang, 2025), which is why the signal needed for opening the K^+^ channel may be delayed or weak.

Also, hyperglycemia affects the dynamics and interactions between the channels: in metabolic stress and under low ATP concentrations, reduced functionality of K_ATP_ channels reduces hyperpolarization, which further reduces the activity of Kv and Kir channels (Wang et al., 2022). Also, the administration of K_Ca_ activators shows that K_Ca_ channels in a DM model have the compensation potential in case there is reduced function of other K^+^ channels (Mishra et al., 2021). T2DM also disrupts the internal networks of proteins that modulate K^+^ channels. For example, oxidative stress reduces the connection of channels with the scaffold proteins by disrupting the cytoskeleton, which changes their local density and the efficacy of hyperpolarization (Capera et al., 2019). Therefore, in DM, the sensitivity of K^+^ channels to different signals, modulatory interactions, and network context is compromised, even before the occurrence of significant structural and functional changes of K^+^ channels, which reflect early stages of vascular dysfunctions.

### 4.1 Expression and functional changes of K^+^ channels in hyperglycemia

In DM, chronic hyperglycemia and metabolic stress trigger complex intrinsic pathways that affect the expression and biokinetics of K^+^ channels in VSMC. In models of T2DM, a decrease in RNA transcripts and protein expression of K_ATP_ channel subunits in VSMC was recorded, which is associated with a decrease in the K_ATP_ current in the conditions of metabolic deficits (Nieves-Cintrón et al., 2021). In a study on human vascular samples, antibodies against Kir6.2 and SUR2B showed weaker signaling in the diabetic internal mammary artery, indicating a change in membrane channel density (Rajkovic et al., 2024). Similarly, in the diabetic human saphenous vein, reduced expression of the SUR2B subunit in the VSMC was observed (Rajkovic et al., 2020).

In BK_Ca_ channels, several studies show not only a decrease in protein expression but also changes in intrinsic sensitivity to Ca^2+^ and voltage (Nieves-Cintrón et al., 2017). In DM, BK_Ca_ channels in arterial smooth muscle require higher levels of Ca^2+^ for activation, which reduces their contribution to vasodilatation.

Kv channels, Kv1 and Kv7, are also subject to restriction: in experimental DM, a decrease in mRNA for Kv7.4 and Kv1.5 subtypes has been observed, which causes a decrease in the amplitude of Kv currents in the VSMC of elastic arteries from high-fat diet mice (Nieves-Cintrón et al., 2015). This accelerates depolarization and reduces resistance to contraction.

A smaller but significant effect on Kir channels was observed in endothelial Kir2.x subtypes. In certain comorbidities, such as hypertension, their currents show reduced amplitude at high extracellular K^+^, which subtly weakens the effect of enhancing hyperpolarization (Do Couto et al., 2024). Similarly, Li et al. reported decreased expression of the K_ATP_ channels and reduced K_ATP_ channel-mediated relaxation in the model of the human umbilical artery of women with GDM (Li et al., 2018). The possible explanation for lower expression of the SUR2B subunit on the K_ATP_ channel in VSMC is explained by the high concentrations of highly reactive species like methylglyoxal (MGO), which led to the mRNA instability of the K_ATP_ channel (Yang et al., 2012). It was suggested that the exposure to the MGO elevates the expression of miR-9a-3p, which subsequently downregulates the SUR2B mRNA, compromising the entire K_ATP_ channel function (Li et al., 2015b).

Functionally, in arterioles from diabetic animals, BK_Ca_ channel agonists trigger noticeably weaker relaxation compared to healthy blood vessels, reflecting a reduced responsiveness of the K^+^ channels (Liu et al., 2024). Also, an experiment with K_ATP_ agonists shows a reduced maximal relaxation in the presence of hyperglycemia, suggesting that the channel may be present but less responsive (Li et al., 2015a).

An important aspect of K^+^ channel regulation in DM is their post-translational modification. Oxidative stress and protein glycation cause damage to Ca^2+^ binding sites in the BK_Ca_ channels (Sun et al., 2024). Changed concentrations of kinases and phosphatases (such as protein kinase C and protein kinase A) favor phosphorylation that reduces K^+^ channel activity (Pan et al., 2022). In kidneys, altered expression of K^+^ channels, especially Kir and K_ATP_, disrupts the blood flow and their glomerular filtration (Guo et al., 2024). All of these changes reduce sensitivity and responsiveness of different vasoactive substances to the K^+^ channels.

### 4.2 Impact of oxidative stress and inflammation on K^+^ channels

ROS and reactive nitrogen species directly modify the structure of K^+^ channels and change their conductance, opening kinetics, and sensitivity to a specific cell membrane potential (Lewandowska et al., 2024). These modifications are caused by oxidation of thiol groups, nitrosylation, or carbonylation of the main amino acids in the transmembrane and regulatory domains of K^+^ channels (Mahapatra et al., 2024).

In VSMCs, oxidative stress decreases the activity of BK_Ca_ and K_ATP_ channels. At the molecular level, ROS inhibit BK_Ca_ channels by oxidizing cysteine residues and damaging beta1 subunits, while simultaneously reducing the sensitivity of K_ATP_ channels to ATP (Liu et al., 2024). These changes increase peripheral resistance.

ECs are particularly sensitive to redox disturbances, as ROS reduce the activity of K_Ca_ channels in the ECs as well (especially K_Ca_2.3 and K_Ca_3.1), thereby reducing EDH and NO release (Schaffer et al., 2012). At the same time, inflammatory cytokines such as tumor necrosis factor alpha (TNF-α) and interleukin 1 beta (IL-1β) decrease the expression of K^+^ channels through the activation of nuclear factor kappa-light-chain-enhancer of activated B cells (NF-κB) and mitogen-activated protein kinase (MAPK) signaling pathways, which further impairs vascular relaxation and ion balance.

Modern researchers indicate that oxidative stress damages mitochondria as well. Loss of functionality of mitochondrial K^+^ channels (especially mitoBK_Ca_ and mitoK_ATP_) increases intracellular ROS production and activates pro-apoptotic signals, which create a vicious circle of oxidative damage (Handy and Loscalzo, 2012; Kulawiak et al., 2023). Studies from 2024 showed that the activation of redox-sensitive pathways, including thioredoxin and glutathione systems, partially protects K^+^ channels from oxidative stress-mediated inhibition and improves vascular reactivity (Hilgers and Das, 2024).

Thus, oxidative stress and inflammation act as central mechanisms that disrupt the fine-tuned balance between depolarizing and hyperpolarizing currents in blood vessels. Understanding these processes provides the basis for the development of new therapeutic strategies that target the restoration of K^+^ channel functionality in states of increased oxidative and inflammatory stress that are present in DM.

### 4.3 The role of K^+^ channels in the development of acute complications of DM

Cerebral edema is a rare and the most serious acute complication of hyperglycemic hyperosmolar (HHS) and ketoacidotic (DKA) coma in DM. Chronic hyperglycemia, oxidative stress, and protein glycation reduce the function of Kir channels in the cerebral arteries and promote vasoconstriction, inflammation, and increased permeability of the blood-brain barrier (Yang and Yang, 2025). Dysfunctional Kir channels permit too much water entry into astrocytes and ECs and lead to vasogenic and cytotoxic edema, especially if the correction of glycemia and osmolarity is too strong (Yuen et al., 2008; Azova et al., 2021). Therefore, selective pharmacological modulation of VSMC K_ATP_ and BK_Ca_ channels in cerebral microcirculation represents a potential method for preventing cerebral edema in patients with DM.

## 5 Pharmacological modulation of VSMC K^+^ channels in DM

The pharmacological modulation of different K^+^ channels may be one of the treatment options for vascular complications in DM. Pharmacological modulators of K^+^ channels include clinically already relevant drugs, agents in preclinical phases of drug development (e.g., iptakalim), and various natural compounds (Gribble and Reimann, 2002). Empagliflozin is a clinically approved SGLT2 (sodium-glucose cotransporter type 2) inhibitor used for the treatment of heart failure primarily in diabetic patients. Mechanistically, it enhances the expression of the BK-β1 subunit in the VSMC and the consequent vasorelaxation of coronary arteries via Silent Information Regulator 1 (Sirt1)/Nuclear-Related Factor 2 (NRF2) signal pathways (Kong et al., 2024). Sulfonylureas are another example of drugs that are in clinical use for treating T2DM. They work by closing the K_ATP_ channels in the pancreatic cells, thereby stimulating insulin release. However, they may close K_ATP_ channels in the VSMCs as well, which reduce vasodilatation and compromise blood flow, which limits their use, especially in patients with different vascular diseases (Pantalone et al., 2010). Another drug, levosimendan (calcium sensitizer and inodilator), is a novel calcitrope used to treat heart failure without increasing intracellular Ca^2+^ concentrations. Mechanistically, it increases the sensitivity of contractile proteins to Ca^2+^ in the cardiac cells. Additionally, it opens mitochondrial and sarcolemmal K_ATP_ channels in cardiomyocytes and VSMC, which is why it is considered a cardioprotective and vasculoprotective agent (Milligan and Fields, 2010).

To overcome the issue with sulfonylureas, pharmacological modulators that simultaneously open VSMC Kir6.1/SUR2B and close pancreatic beta-cell Kir6.2/SUR1 K_ATP_ channels are being developed. One of those agents is iptakalim, which is currently in preclinical investigation as a drug for T2DM with an additional vasorelaxant property. Natural compounds and plant extracts such as flavonoids, polyphenols, and alkaloids can directly or indirectly modulate BK_Ca_, K_ATP_ channels, and Kv channels, leading to cell membrane hyperpolarization and vasodilatation. Among those compounds, the most promising results are shown for quercetin, resveratrol, and polyphenols from green tea, the latter of them having additional endothelial-protective and anti-inflammatory properties (da Silva et al., 2025). Ginsenosides RE from *Panax ginseng* modulate SK_Ca_ channels in the ECs of coronary arteries, improving blood flow (Kim, 2012). While these natural modulators show multiple beneficial effects (anti-inflammatory, antioxidative, vasorelaxant, and endothelial protection), most evidence comes from *in vitro* or animal studies. Translational studies are needed to define optimal doses, extract standardization, precise mechanisms of action, and long-term safety in humans.

The concept of targeted-channel therapy in clinical settings is getting closer to reality. Some of those agents (such as empagliflozin) are widely in current clinical use, but there are many experimental therapies that are under preclinical or early translational investigation. Positive results from extensive research on the long-term efficacy and safety of K-targeted drugs in the treatment of people with T2DM are expected in the future.

## 6 Conclusion and future perspectives

The increasing number of DM patients with vascular complications requires special and up-to-date treatment. K^+^ channels stand out as the important molecular target for the development of new and innovative therapies. Currently available data support further research and the development of drugs and solutions that target K^+^ channels in the blood vessels, which could benefit all patients with vascular diseases, including the patients with DM.

While conceptually attractive, this approach is highly context-dependent, varying by channel subtype, vascular bed, and disease stage. However, structural and functional changes in K^+^ channels in DM-associated vascular complications open up new avenues for novel therapy. Based on all of the above, those may be, for example, activators of K^+^ channels, novel antidiabetics with pleiotropic effects, or natural compounds, like red wine polyphenol resveratrol. Our next review will further evaluate benefits and pitfalls for optimizing novel K^+^ channel-targeted therapy and its integration into standard healthcare for vascular complications of DM.
